# Snowmelt predicts earlier breeding across the latitudinal range of an Arctic nesting seabird, the Little Auk (*Alle alle*)

**DOI:** 10.1111/1365-2656.70287

**Published:** 2026-06-03

**Authors:** Martyna Syposz, Øystein Varpe, Sébastien Descamps, Jérôme Fort, David Grémillet, Ann Harding, Dariusz Jakubas, Dorota Kidawa, Nomikos Skyllas, Hallvard Strøm, Tom S. L. Versluijs, Katarzyna Wojczulanis‐Jakubas

**Affiliations:** ^1^ Department of Vertebrate Ecology and Zoology, Faculty of Biology University of Gdańsk Gdańsk Poland; ^2^ Department of Biological Sciences University of Bergen Bergen Norway; ^3^ Norwegian Institute for Nature Research (NINA) Bergen Norway; ^4^ Norwegian Polar Institute, Fram Centre Tromsø Norway; ^5^ Littoral Environnement et Sociétés (LIENSs), UMR 7266 La Rochelle University‐CNRS La Rochelle France; ^6^ CEFE, Université Montpellier, CNRS, EPHE, IRD Montpellier France; ^7^ FitzPatrick Institute of African Ornithology, DST‐NRF Centre of Excellence University of Cape Town Cape Town South Africa; ^8^ Auk Ecological Consulting Cordova Alaska USA; ^9^ Institute of Oceanology, Polish Academy of Sciences Sopot Poland; ^10^ Energy and Sustainability Research Institute Groningen (ESRIG) University of Groningen Groningen The Netherlands; ^11^ Royal Netherlands Meteorological Institute (KNMI) De Bilt The Netherlands; ^12^ Department of Theoretical and Computational Ecology Institute for Biodiversity and Ecosystem Dynamics, University of Amsterdam Amsterdam The Netherlands; ^13^ NIOZ Royal Netherlands Institute for Sea Research Den Burg The Netherlands; ^14^ University of Groningen Groningen The Netherlands

**Keywords:** chick survival, climate change, dovekie, High Arctic, phenotypic plasticity, population dynamics, seasonality, trophic mismatch

## Abstract

1. Climate‐driven temporal shifts in seasonal environments are altering some of the environmental cues that organisms use to time reproduction, potentially leading to trophic mismatches across ecosystems. In the Arctic, marine predators must balance conditions at sea with local terrestrial constraints at breeding sites, yet the relative importance of these cues for breeding phenology remains unclear.
2. We used a crevice‐nesting High‐Arctic planktivorous seabird, the Little Auk (*Alle alle*), as a model species. Drawing on a unique multi‐year dataset from four colonies with distinct climatic regimes, we tested whether breeding onset tracks the timing of snowmelt at breeding sites, a key terrestrial cue determining nest accessibility.
3. Snowmelt timing is closely linked with hatching date, with earlier snowmelt enabling earlier access to nesting crevices and advancing hatching across all sites.
4. Importantly, we detected no significant directional temporal trend in snowmelt timing over the study period (2000–2024), suggesting that this relationship reflects interannual variability.
5. Within years, later hatching was associated with reduced chick growth and survival across colonies. However, interannual variation in mean hatching date was linked to chick survival in only one colony, indicating spatial heterogeneity in demographic consequences of breeding phenology.
6. Future projections indicate that snowmelt timing will advance where little auks breed, potentially advancing breeding timing. However, other ongoing changes—such as borealization of zooplankton communities and the loss of summer sea ice—may alter future fitness consequences of breeding timing.
7. Our study highlights the role of the terrestrial environment in shaping the breeding timing of high‐latitude marine birds.

## Graphical abstract

Multi‐year monitoring of the crevice‐nesting High Arctic seabird, the Little Auk (*Alle alle*), across four colonies spanning distinct climatic regimes revealed that snowmelt timing is a key and consistent driver of breeding phenology. Earlier snowmelt advances access to nesting habitat, enabling birds to initiate reproduction sooner. These findings show how rapid environmental change in the Arctic reshapes breeding schedules and suggest that phenological plasticity may allow some populations to initially benefit from earlier snowmelt. Graphics by Karolina Cieślińska.

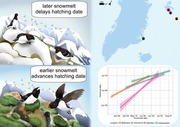

## 1 INTRODUCTION

The annual cycle of solar radiation drives seasonal variations, characterized by a distinct temporal window of habitat availability and primary production (Ji et al., 2013). In response to this seasonality, organisms have evolved schedules for breeding and other life‐history events to align with periods of optimal environmental conditions (Samplonius et al., 2021; Varpe, 2017). However, climate change may disrupt these relationships, leading to a variety of alterations in food web dynamics (Thackeray et al., 2016; Visser, 2008). As different trophic levels often require specific conditions for reproduction, climate‐induced shifts can decouple predator–prey interactions, potentially resulting in reduced reproductive success and fitness in consumers (Cushing, 1990; Hjort, 1914; Thomas et al., 2001). This disruption in synchrony is commonly referred to as the ‘mismatch hypothesis’ (Cushing, 1990). For example, the reproductive success of many predators depends on their prey availability during the breeding season, which in turn is closely linked to climatic conditions (e.g. Kitaysky & Golubova, 2000; Visser et al., 1998).

The Arctic is particularly vulnerable to climate change, warming at more than twice the global average and with significant sea ice loss (IPCC, 2023). Precipitation, either snow or rain, is also increasing across the region and projections suggest this trend will continue (Førland et al., 2011). Different forms of precipitation have contrasting effects on the snowpack: while snowfall can temporarily delay melt by increasing snow depth and surface albedo, rain and rain‐on‐snow events can accelerate melting by adding heat and altering the snow structure. Overall, while there is substantial regional variability, the duration of snow cover is decreasing across much of the Arctic (Liston & Hiemstra, 2011). This shift has led to earlier breeding and changes in the reproductive success in many ground‐nesting species. For example, Pink‐footed Geese (*Anser brachyrhynchus*) initiate breeding earlier in warmer years, with up to a five‐fold increase in reproductive success (Madsen et al., 2007). In contrast, Brünnich's Guillemots (*Uria lomvia*) also breed earlier, but it is hypothesized that this shift is insufficient to match changes in food availability, leading to poorer conditions for their offspring (Gaston et al., 2009; Whelan et al., 2022). While climate change alters wildlife population dynamics in the Arctic (Grémillet & Descamps, 2023), the direction and magnitude of these changes remain generally difficult to predict (e.g. Bårdsen et al., 2011).

The little auk (or dovekie, *Alle alle*), the most numerous Arctic seabird species, exemplifies the complex interplay between climate change and seabird life‐history. Indirect effects occur through borealization, whereby lipid‐rich Arctic copepods are increasingly replaced by smaller species. Because little auks forage selectively on these Arctic copepodes, such changes may impose significant energetic constrains (Descamps et al., 2022; Renaud et al., 2018). Yet, while some studies conducted over the past decades on various little auk populations have reported decreasing survival, breeding success, or population size in association with ocean warming (Descamps et al., 2022; Hovinen, Welcker, et al., 2014; Jakubas et al., 2022, 2024; Kidawa et al., 2015; Sauser et al., 2023), others have documented behavioural plasticity and potential for energetic compensation, with survival and fecundity maintained at past levels (e.g. Amélineau et al., 2016; Beaman et al., 2024; Grémillet et al., 2012; Jakubas et al., 2020). Little auks are also observed to breed earlier in years with a warm spring (Moe et al., 2009). It was hypothesized that little auk nests located between crevices are blocked by snow and only when it melts, seabirds can start breeding (Jakubas & Wojczulanis‐Jakubas, 2011; Moe et al., 2009). Such terrestrial effects on breeding timing have also been hypothesized for other high‐latitude seabird species (Burr et al., 2016). However, it remains unclear whether this advancement in breeding phenology is driven by changes in snow conditions and whether earlier breeding occurs uniformly across different populations of the species. In this context, fitness consequences of earlier breeding of the little auk also remain unknown.

Here we combine long‐term data on little auk breeding biology from four breeding colonies across a latitudinal gradient that includes contrasting oceanographic and climatic regimes. The aim of this study is to test whether little auks' breeding phenology is correlated with the timing of snowmelt at the colony site. To establish the moment when nest sites in rock crevices are available and breeding could start, we used day of snowmelt in the colony (Jakubas & Wojczulanis‐Jakubas, 2011; Moe et al., 2009). Additionally, we examined how alterations in breeding onset affect reproductive success, predicting that changes in the timing of breeding may influence chick growth and survival rates. Finally, to place our findings in a future climate context, we analysed CMIP6‐based projections of Arctic surface snow cover across three emissions scenarios, aiming to assess potential long‐term changes in snowmelt timing at little auk colonies.

## 2 MATERIALS AND METHODS

### 2.1 Species, study site and available data

**FIGURE 1:**
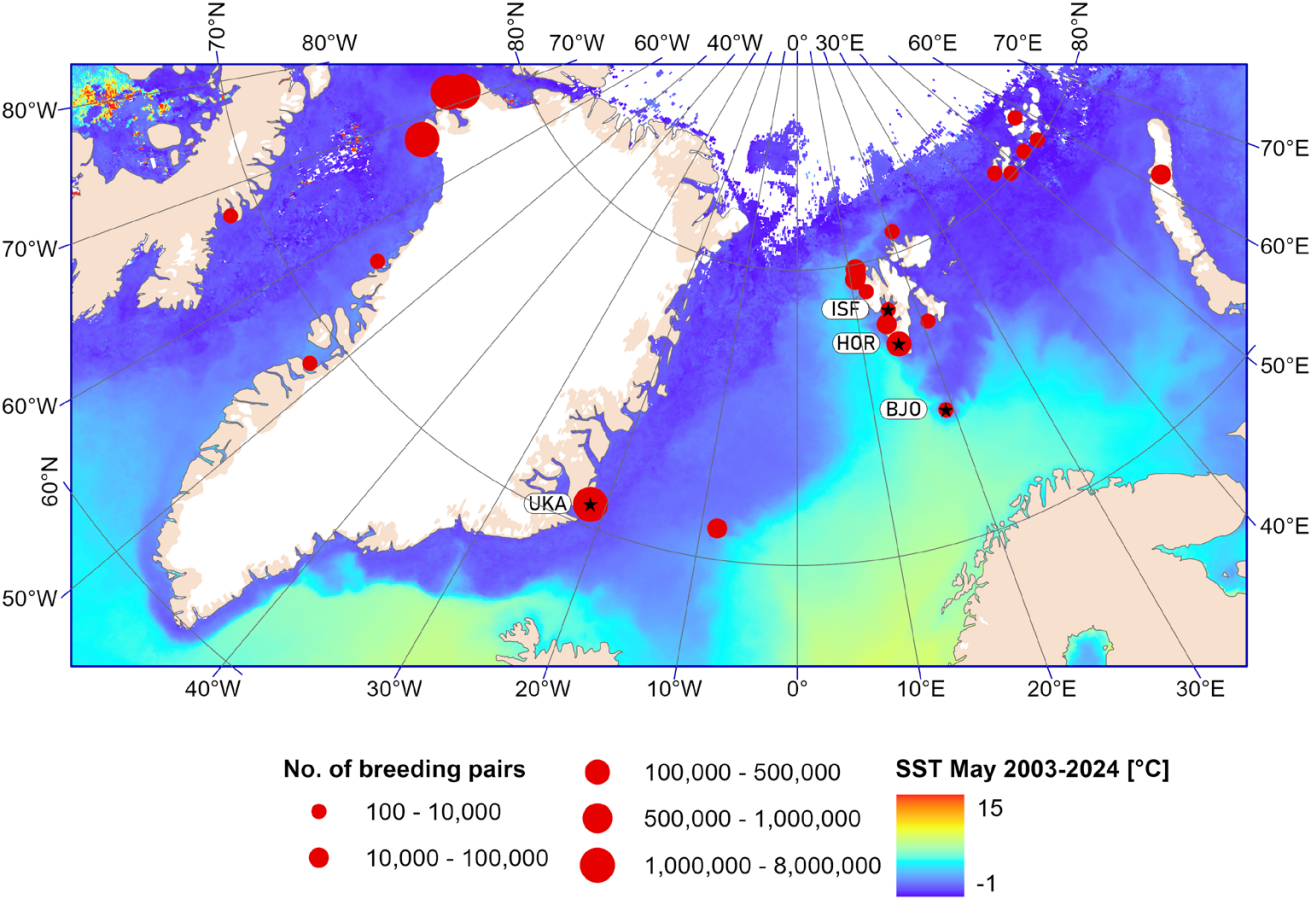
Locations of the breeding colonies of little auks in the North Atlantic (red circles). The four study colonies are highlighted as black stars (ISF—Isfjorden, HOR—Hornsund, BJO—Bjørnøya, UKA—Ukaleqarteq) and represent some of the largest and latitudinally most distant colonies (estimated colony sizes after Keslinka et al., 2019). Background: Mean SST in May 2003–2024; *Source:* Moderate‐resolution imaging spectroradiometer (MODIS) Aqua satellite data from Ocean Colour Data webpage, https://oceancolor.gsfc.nasa.gov/). Map in polar stereographic projection was produced in ArcMap 10.3.1 (Redlands, CA: Environmental Systems Research Institute). Base map: Natural Earth (@naturalearthdata.com).

The little auk is the smallest, most numerous seabird breeding in the High Arctic with its numbers estimated at 40–80 million individuals (BirdLife International, 2018). Given its abundance, its feeding ecology, and transport of large amounts of organic matter from sea to land, fertilizing the nutrient‐deprived Arctic tundra (González‐Bergonzoni et al., 2017), the little auk is considered an important species for the functioning of High Arctic marine and terrestrial ecosystems (Stempniewicz et al., 2007; Wojczulanis‐Jakubas et al., 2022). The large, lipid‐rich (energy‐rich) cold water copepods (*Calanus glacialis* and *C. hyperboreus*) are the main prey of little auks (Frandsen et al., 2014; Kwasniewski et al., 2010). The little auk is also one of the few crevice‐nesting seabirds in the North Atlantic. The data in the present study were collected in Ukaleqarteq (Kap Høegh; 70°44′ N 21°35′ W) in East Greenland and three colonies in the Svalbard archipelago: Bjørnøya (74°23′ N 19°2′ E), Hornsund (77°1′ N, 15°32′ E) and Isfjorden (78°13′ N, 15°19′ E, Figure 1).

In all the locations, a sample of little auk nests are regularly monitored to assess their hatching dates. For the purpose of the present study, we combined hatching date data spanning about 20 years (2004–2024, Supporting Information 1, Table S1, Figure S1). While egg laying date would have been the most accurate approximation of breeding onset, these data were available only for a few years at the Hornsund colony due to logistical constraints of Arctic fieldwork. For these data median laying date is highly correlated with median hatching date (Spearman's correlation, *r* = 0.99, *p* < 0.001; Supporting Information 2, Figure S2). This finding is unsurprising, given the generally extremely consistent incubation durations in birds, driven by strong morphophysiological constraints on embryonic development (Ricklefs, 2006). We therefore proceeded with hatching dates as a proxy for the onset of breeding in all colonies. We included only hatching dates estimated with a precision of ±3 days or less, based on nest monitoring frequency. This threshold ensured a minimum level of temporal accuracy for phenological comparisons. Years with fewer than 10 such nests were excluded from the analysis (Supporting Information 1, Table S1, Figure S1). The data on hatching date were available for 20, 19, 17 and 12 years for Hornsund, Bjørnøya, Ukaleqarteq and Isfjorden, respectively (Supporting Information 1, Table S1). Across colonies, the difference between the earliest and latest median hatching dates across years ranged from 8 days to 12 days.

All field work was conducted in accordance with guidelines for the use of animals. Fieldwork on Bjørnøya, in Isfjorden and Hornsund, Svalbard was conducted under annual permits issued by the Governor of Svalbard and the Norwegian Food Safety Authority (most recent permits relevant to this study: ref. 21/03599‐29, ref. 16/00650‐38 and ref. 23/00904‐21 for Bjørnøya, Isfjorden and Hornsund, respectively). Experiments in Ukaleqarteq were annually approved by the Danish Polar Center and the Government of Greenland, Ministry of Environment and Nature and Department of Fisheries, Hunting and Agriculture (most recent permit relevant to this study: N° 2023‐6417).

### 2.2 Quantifying the date of snowmelt using MODIS

All analyses were done in R software version 4.5.1 (R Development Core Team, 2025).

We calculated the date of snowmelt for each study site and year based on near‐daily satellite images from the 500 m MODIS Terra Surface Reflectance Daily Global product (MOD09GA, v6.1, Vermote & Wolfe, 2021). We thereby followed the automated workflows by Versluijs (2025), using the *rgee* package (Aybar et al., 2020) in R software and Google Earth Engine (Gorelick et al., 2017).

For each study site, we first constructed a polygon covering the spatial extent of the breeding colony (Supporting Information 3, Table S2). We subsequently extracted MODIS satellite images for each polygon between March 15th and September 15th for all years from 2000 to 2024. This time window includes the whole breeding season of little auks and interannual and inter‐colony variability in snowmelt. We masked pixels from the analysis if they were classified as cloud covered by the MOD35 cloud mask product (Ackerman et al., 2015). To quantify snow cover within each polygon, we calculated the average normalized difference snow index (i.e. NDSI; Dozier, 1989; Dietz et al., 2012) among all pixels in each extracted image, by taking the normalized difference between band 4 (Green) and band 6 (short‐wave infrared, SWIR):

$$\text{NDSI}=\frac{\left({\text{Green}}_{B04}-{\text{SWIR}}_{B06}\right)}{\left({\text{Green}}_{B04}+{\text{SWIR}}_{B06}\right)}$$

To extract the annual date of snowmelt per polygon we fit GAMs (generalized additive models; Wood, 2023) to the annual NDSI timeseries per polygon, using the default thin plate regression splines from the *mgcv* package and using a two‐step outlier filtering approach (Supporting Information 4). As NDSI values larger than 0.4 generally correspond to snow‐cover (Dozier, 1989; Hall et al., 1995) we extracted the date when the GAM‐predicted values (smoothed) NDSI values dropped below this snow‐cover presence threshold (Figure S3).

Because each study site was represented by only one to three MODIS pixels (500 m resolution), we validated our snowmelt estimates against higher resolution Sentinel‐2 data (20 m, 2019–2024), which confirmed comparable interannual trends (Supporting Information 5, Figures S4 and S5).

### 2.3 Projections of Arctic surface snow cover

We analysed projected changes in surface snow cover area fraction (variable ‘snc’, defined as the percentage of a grid cell covered by snow) using simulations from the Coupled Model Intercomparison Project Phase 6 (CMIP6) proposed by Eyring et al. (2016). We considered three Shared Socioeconomic Pathways (SSPs): SSP1‐2.6, SSP2‐4.5, and SSP5‐8.5, which represent alternative greenhouse gas emissions trajectories based on different socioeconomic futures. These scenarios are commonly used in climate modelling to explore a range of potential climate outcomes, from strong mitigation (SSP1‐2.6) to intermediate (SSP2‐4.5) and high emissions (SSP5‐8.5). In summary, they were used because they span a wide range of possible outcomes and are the most frequently used subset. Our analysis was based on output from nine Earth System Models (ESMs): CanESM5, CMCC‐CM2‐SR5, CMCC‐ESM2, INM‐CM4‐8, INM‐CM5‐0, IPSL‐CM6A‐LR, MIROC6, MPI‐ESM1‐2‐HR, and MPI‐ESM1‐2‐LR. Model data were obtained from the Earth System Grid Federation (ESGF).

Daily snow cover outputs were retrieved from both the historical and scenario simulations and merged to form continuous time series from 1850 to 2100. To ensure a consistent spatial domain, all model outputs were converted to a common 1° × 1° regular grid using bilinear interpolation. We then extracted the Arctic region (60°–90° N) and applied a series of spatial masks to isolate ecologically relevant land pixels. These masks were derived from high‐resolution ERA5‐based datasets and converted to match spatially the climate model grid. To approximate suitable habitat in CMIP6 outputs, we applied standard spatial masks combining land–sea separation, vegetation cover, and elevation filters. It is important to note that satellite‐based and climate model‐based snow cover datasets differ substantially in spatial resolution and processing methods. Satellite observations offer high‐resolution data (typically ~500 m), allowing precise analysis of small areas. In contrast, CMIP6 model outputs have much coarser resolution (~100 km), necessitating the use of broader spatial masks to approximate relevant habitats. These 1° × 1° masks were applied to increase the ecological relevance of our model projections, particularly for species such as the little auk.

We conducted all climate model postprocessing and mask operations using the Climate Data Operators (CDO) software (Schulzweida, 2023). For each SSP, we calculated ensemble means and standard deviations across the nine models. Snow cover values were aggregated to yearly and monthly means, and all analyses were limited to the period 2000–2100. For regional analyses, we subset the data to East Greenland and Svalbard, corresponding to key case study areas in our ecological analyses.

The CMIP6 analyses are intended to provide regional‐scale climate context rather than colony‐specific forecasts of breeding phenology. Given the coarse spatial resolution (~1°) and methodological differences between model‐derived snow cover fraction (snc) and the high‐resolution satellite‐derived snowmelt timing used in the phenological analyses, we do not directly translate CMIP6 outputs into quantitative projections of hatching date or demographic parameters. Instead, these projections are used to illustrate the magnitude, direction, and spatial heterogeneity of expected changes in Arctic snow cover across key breeding regions.

### 2.4 Statistical analysis

We used linear mixed‐effect models (LMMs) using the *lme4* package (Bates et al., 2015) to examine the relationship between the hatching date of little auk and snowmelt (see Table 1). Our explanatory variables included snowmelt day at the colony as continuous variables, location as a categorical variable, and interaction between them. We included random intercepts for nest identity and colony–year to account for repeated measurements and non‐independence among observations. To improve the interpretability of interaction terms, snowmelt date was centred within colonies prior to analysis. We checked for multicollinearity by calculating the Variance Inflation Factor (VIF) for each term using the *car* package (Fox et al., 2007), and none of the terms were above 3. We also checked model assumptions of residual heteroscedasticity and normality by inspecting residual distribution and tested for temporal autocorrelation in the residuals.

**TABLE 1:** Overview of statistical models used in the study.

Model name	Response variable	Fixed effects	Random effects	Error distribution
Linear mixed‐effects model	Hatching date (DOY)	Colony‐centred snowmelt date × Location	Nest; Location × Year	Gaussian
Linear mixed‐effects model	Snowmelt date (DOY)	Year × Location	Location	Gaussian
Linear mixed‐effects model	Chick growth rate	Within‐year hatching date + Between‐year mean hatching date	Year	Gaussian
Generalized linear mixed‐effects model	Chick survival (0/1)	Within‐year hatching date + Between‐year mean hatching date	Year	Binomial (logit link)

To test for temporal trends in snowmelt from years 2000–2024, we modelled annual snowmelt days as a function of year (mean‐centred), location, and their interaction using Linear Mixed‐Effects Models. Year was mean‐centred (year − mean(year)) prior to analysis to improve interpretability of model parameters and reduce collinearity between main effects and interaction terms. Location was included as a fixed effect to test for differences among colonies, while a random intercept for location was included to account for repeated measurements within colonies. Location‐specific slopes were extracted with the *emmeans* package and p‐values adjusted for multiple testing (Lenth et al., 2018).

#### 2.4.1 Chick variables

We investigated whether chick growth rate and survival were related to the onset of breeding. These data were available for Hornsund (chick growth and survival), Ukaleqarteq (chick growth) and Isfjorden (survival). Chick survival was assessed for the first 15 days of life—this period is considered critical, as chicks are particularly vulnerable to food deprivation (Gębczyński et al., 1996; Taylor & Konarzewski, 1989). Additionally, while predation can occur at any time, disappearances during the first 2 weeks are easier to identify during routine nest checks. During this period, chicks remain inside the nest chamber, and signs such as the absence of the chick, polar fox (*Vulpes lagopus*) scent or fur at the nest entrance, and/or displaced rocks provide clear evidence of predation. After day 15, chicks may begin to leave the chamber to exercise their wings and may be predated outside the nest—often by polar foxes or glaucous gulls (*Larus hyperboreus*)—without leaving obvious traces. As variables reflecting chick growth rate, we chose the slope of the first segment of the chick growth curve (the rapid and linear increase in chick body mass), which is up to the 15th day of chick life, and included chicks that were weighed at least 3 times during this period. We analysed the effect of hatching date on chick survival using generalized linear mixed‐effects models (GLMMs; binomial error distribution with logit link) and on chick growth using linear mixed‐effects models (LMMs). In both cases, hatching date was decomposed into a within‐year component (deviation from the annual mean) and a between‐year component (mean annual hatching date) to distinguish relative timing effects from interannual variation. Year was included as a random intercept to account for additional interannual variation in environmental conditions influencing chick performance.

## 3 RESULTS

### 3.1 Environmental predictors of hatching date

**FIGURE 2:**
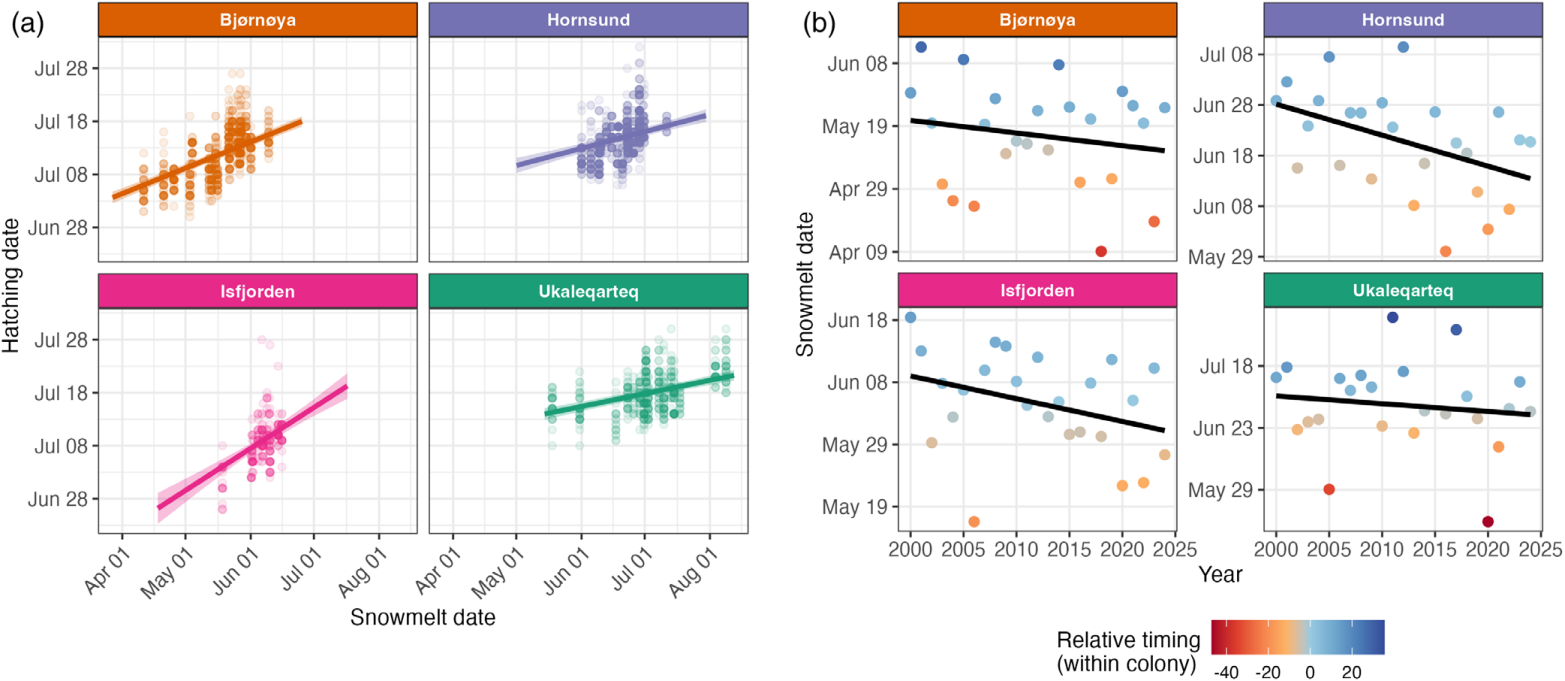
(a) Relationship between snowmelt date and hatching date for four colonies of little auks. Solid lines indicate colony‐specific fits from LMM, which included an interaction between colony and snowmelt date. Shaded areas represent 95% confidence intervals. (b) Temporal variation in snowmelt date at four little auk breeding colonies between 2000 and 2024. Points represent annual snowmelt dates, coloured relative to each colony's mean snowmelt timing (the redder the earlier, the bluer the later). Black lines show fitted linear trends from LMM (all *p* > 0.12).

Earlier snowmelt at breeding sites was consistently associated with earlier hatching across all colonies, confirming that snowmelt timing is a key environmental cue for breeding phenology in little auks. To account for variation among colonies, we included an interaction between colony and snowmelt date. The effect of snowmelt was significant and positive in all colonies (Figure 2a): Bjørnøya (slope: 0.21 ± 0.04, *t* = 6.01, *p* < 0.001), Ukaleqarteq (slope: 0.09 ± 0.03, *t* = 3.19, *p* = 0.00142), Hornsund (slope: 0.19 ± 0.05, *t* = 3.54, *p* < 0.001), Isfjorden (slope: 0.37 ± 0.1, *t* = 3.92, *p* < 0.001).

This means that a 10‐day advance in snowmelt corresponds to a 3.7‐day advance in hatching in Isfjorden, compared to just 0.9 days in Ukaleqarteq. However, this relationship was driven by interannual variability in snowmelt timing rather than a consistent long‐term trend, as there were no significant directional changes in snowmelt timing at any of the colonies (Figure 2b): Bjørnøya (slope = −0.40 ± 0.38 days year^−1^, *t* = −1.07, *p* = 0.288), Hornsund (−0.61 ± 0.38, *t* = −1.63, *p* = 0.107), Isfjorden (−0.37 ± 0.38, *t* = −0.97, *p* = 0.335), and Ukaleqarteq (−0.32 ± 0.38, *t* = −0.83, *p* = 0.407).

### 3.2 Chick variables

**FIGURE 3:**
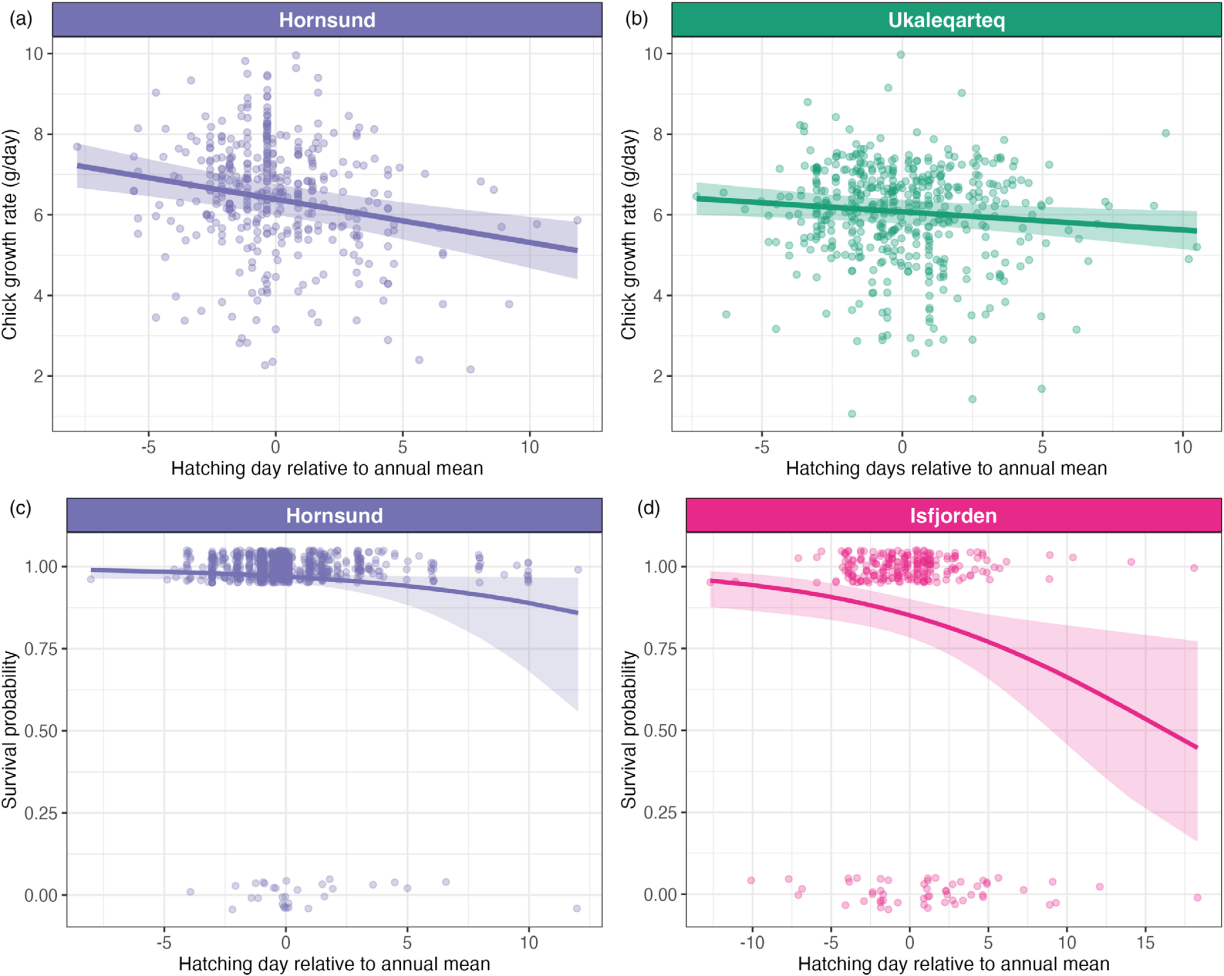
Predicted relationships between relative hatching date (days from the annual mean; negative values indicate earlier‐than‐average broods, positive values indicate later‐than‐average broods) and chick growth rate (a, b) or survival to day 15 (c, d). Solid lines show model predictions, and shaded areas represent 95% confidence intervals. Points show observed chick level data.

Chick performance declined with later hatching date within years across colonies. In Hornsund, chicks that hatched later than the annual mean exhibited slower growth (LMM: −0.123 ± 0.027, *t* = −4.53, *p* < 0.001; Figure 3a) and lower survival to day 15 (GLMM: −0.138 ± 0.065, *z* = −2.13, *p* = 0.034; Figure 3c). Similarly, in Ukaleqarteq, later‐than‐average chicks grew more slowly (LMM: −0.045 ± 0.019, *t* = −2.37, *p* = 0.018; Figure 3b), and in Isfjorden, later hatching within a year was associated with reduced survival (GLMM: −0.107 ± 0.040, *z* = −2.68, *p* = 0.007; Figure 3d). These results indicate consistent directional selection favouring earlier breeding within years.

In contrast, interannual variation in mean hatching date showed limited associations with chick performance. Mean annual hatching date was not related to chick growth in Hornsund (0.011 ± 0.069, *t* = 0.16, *p* = 0.877) or Ukaleqarteq (−0.058 ± 0.055, *t* = −1.06, *p* = 0.307), nor to chick survival in Hornsund (−0.062 ± 0.091, *z* = −0.68, *p* = 0.496). However, in Isfjorden, years characterized by later mean hatching dates exhibited lower chick survival (−0.296 ± 0.114, *z* = −2.60, *p* = 0.009), indicating that population‐level advancement of breeding was associated with improved survival in this colony.

### 3.3 Projections of Arctic surface snow cover

**FIGURE 4:**
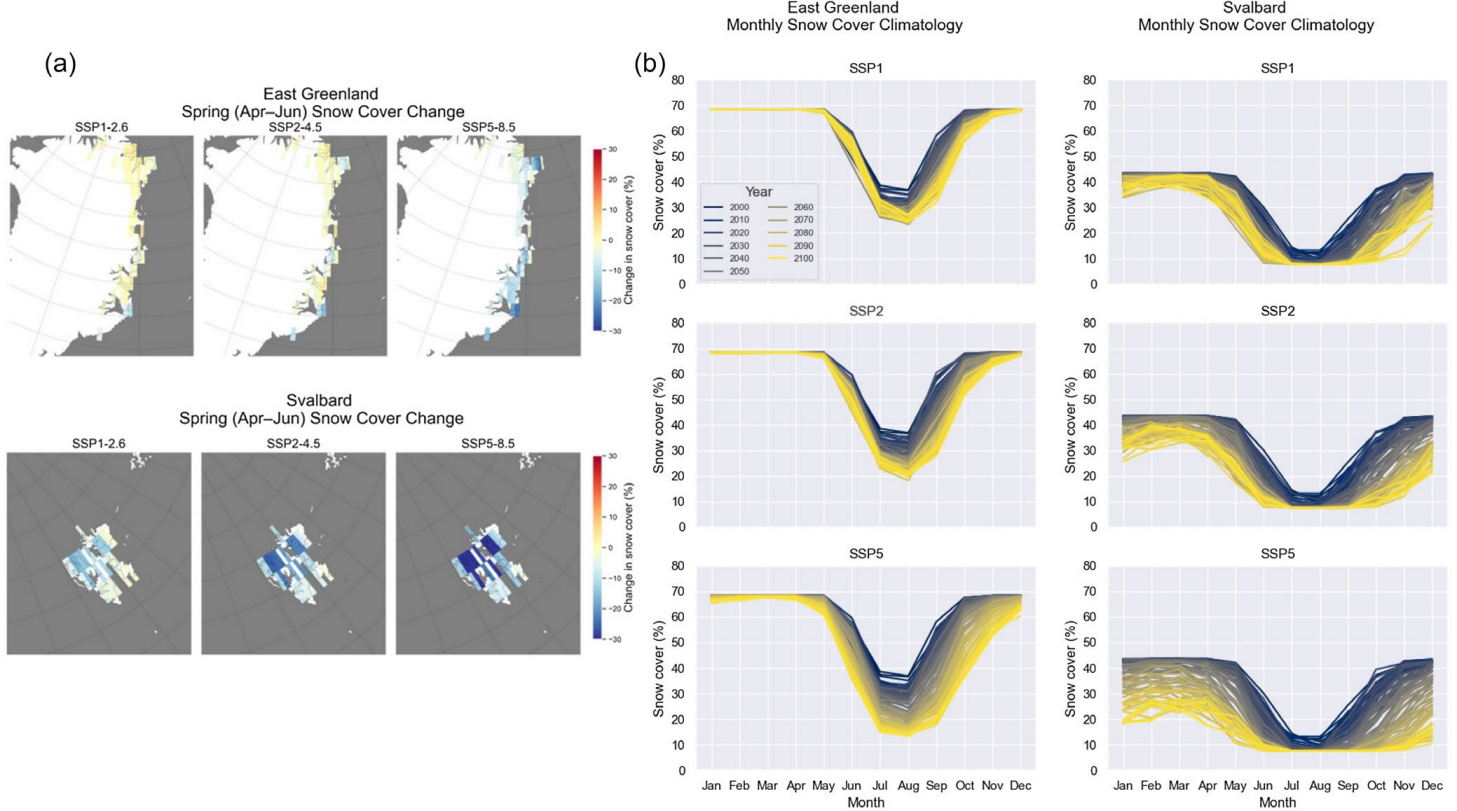
(a) Change in spring (April–June) mean snow cover (%) per grid cell between the future (2080–2099 average) and the recent past (2000–2019 average), for the two regions studied (rows) and the three SSP scenarios (columns). Positive values (red) indicate increased snow cover, while negative values (blue) indicate a decrease. (b) Interannual variation in monthly snow cover for the two study regions (columns) under three SSP scenarios (rows). Each line represents a single year from 2000 to 2100, with line colour indicating time: Dark blue for early‐century and yellow for late‐century. Note that snow cover does not reach 0% because CMIP6 snow cover represents grid‐cell snow fraction and includes perennial snow and glacier ice in some cells.

At the regional scale, CMIP6 projections indicate substantial changes in seasonal snow cover across key breeding regions, providing climatic context for the phenological relationships described above. Our analysis showed that spring snow cover in East Greenland, a proxy for snowmelt timing, decreases primarily under the SSP5‐8.5 scenario, and this trend was most pronounced in the central part of the study region (Figure 4a). In contrast, the northern parts of East Greenland show smaller changes, with snow cover decreasing only slightly over time. In East Greenland, the snow cover—particularly during the April to June period—has begun to decline earlier in spring, while the onset of snow accumulation in autumn is progressively delayed (Figure 4b).

In comparison, Svalbard exhibits the most dramatic changes across all three climate scenarios, particularly in the northern part of the archipelago (Figure 4a). This pattern is consistent with the monthly climatology, which highlights Svalbard's heightened sensitivity to climate warming (Figure 4b). The most pronounced reduction in snow cover was observed in autumn, indicating a delayed onset of snow accumulation and an overall extension of the snow‐free season. These projections describe large‐scale shifts in seasonal snow dynamics; given their spatial resolution, they are not intended to provide precise colony‐level forecasts but rather to highlight regional contrasts and the broader seasonal restructuring of Arctic snow cover.

## 4 DISCUSSION

Our findings demonstrate that the timing of snowmelt is closely linked to the initiation of breeding in little auks across all four studied populations. By using longer time series, a more direct environmental variable, and a broader geographical scope than previous studies (Jakubas & Wojczulanis‐Jakubas, 2011; Moe et al., 2009), our results confirm that snowmelt timing is closely associated with breeding onset. Consequently, the projected advancement of spring snowmelt (Figure 4) is likely to drive shifts in breeding phenology across the species' range in the future.

Earlier hatching within years was associated with faster chick growth and higher survival probability across colonies (Hornsund, Ukaleqarteq and Isfjorden), indicating consistent fitness advantages for individuals breeding earlier than the annual mean. This pattern suggests directional selection favouring earlier breeding within seasons. Similar within‐year advantages of earlier breeding have been reported in other seabird systems (e.g., Hanssen et al., 2013; Madsen et al., 2007; Sauve et al., 2019). However, we found limited evidence that interannual advancement of mean breeding date universally improves chick performance: earlier breeding years were associated with higher survival in only one colony (Isfjorden). This spatial heterogeneity indicates that while earlier relative timing within a season is advantageous, population‐level phenological shifts may not consistently translate into demographic benefits. Future research should therefore examine whether the apparent short‐term within‐year benefits of earlier breeding persist under continued climate warming and across additional demographic components, such as recruitment and adult survival.

Seasonally pulsed snowmelt and temperature at higher latitudes are closely linked to phenology in many polar systems (Assmann et al., 2019; Doiron et al., 2015), including ground‐nesting birds (Dickey et al., 2008; Lameris et al., 2018; Meltofte et al., 2007; Sauve et al., 2019). Given that little auks spend their winter in subarctic waters, they arrive early (April/May) at the colony (Syposz et al., in prep) so they can take advantage of earlier snowmelt. Prior to egg laying, snow might constrain access for little auks to suitable nesting locations in rock crevices (Jakubas & Wojczulanis‐Jakubas, 2011; Moe et al., 2009; Norderhaug, 1980; Stempniewicz, 1981). Additionally, since maintaining an egg at an optimal temperature necessitates time and energy from a bird at the expense of other activities, ensuring that the egg does not lie on snow‐covered ground is especially important for birds living in cold environments (Stevenson & Bryant, 2000). It is possible that females delay their ovulation up until ‘the right moment’, as has been found in Least Auklet (*Aethia pusilla*), Parakeet Auklet (*Aethia psittacula*) and Crested Auklet (*Aethia cristatella*), closely related alcid species (Sealy, 1975). In the little auk, onset of ovulation may be related to direct access to the nest as well as air temperature and other weather conditions, and then regulated behaviourally, with the presence/absence of conspecifics in the colony dependent on environmental conditions. While in the colony, frequent copulations may stimulate female ovulation; indeed, little auk colony attendance and frequency of the copulations intensify over the mating period (Wojczulanis‐Jakubas et al. 2009). Although breeding phenology tracked interannual variation in snowmelt timing, the response was shallower than a 1:1 relationship, indicating partial tracking. In Isfjorden, which showed the strongest predicted response, a 10‐day earlier snowmelt is associated with a 3.7‐day earlier hatching date. This suggests that breeding phenology may be maintained within a relatively narrow window, potentially reflecting both limited capacity for rapid evolutionary change in this long‐lived species and additional ecological constraints, such as prey phenology.

Previous studies on little auks have shown that increased SST is linked to reduced chick body mass, lower chick survival or fledging probability, and altered foraging behaviour (Hovinen, Welcker, et al., 2014; Hovinen, Wojczulanis‐Jakubas, et al., 2014; Jakubas et al., 2020; Kidawa et al., 2015). We found, however, limited support for consistent between‐year demographic benefits of earlier breeding: later hatching date was associated with improved chick survival in only one colony (Isfjorden). This spatial heterogeneity suggests that while individual‐level timing within a season strongly influences chick performance, population‐level advancement of breeding does not universally translate into higher fitness. This aligns with other studies showing that adult little auks are capable of adjusting their provisioning behaviour to varying at‐sea conditions (Amélineau et al., 2016; Grémillet et al., 2015; Jakubas et al., 2017; Jakubas, Wojczulanis‐Jakubas, et al., 2016). Additionally, it is possible that higher air temperatures during the chick‐rearing period result in reduced chick and adult thermoregulation costs and decreased brooding time, thus positively affecting chick vital rates (Beaman et al., 2024; Klaassen et al., 1989; Kulaszewicz & Jakubas, 2018; Lameris et al., 2022; McKinnon et al., 2013), but see (Grunst et al., 2023). Therefore, even if there is asynchrony between little auk breeding period and peak abundance of their prey species, it may be mitigated by positive effects of rising temperature and/or foraging on alternative prey (Jakubas, Wojczulanis‐Jakubas, et al., 2016), suggesting lack of evidence for a trophic mismatch (Samplonius et al., 2021). We, however, tested only two fitness properties at three colonies and other consequences, such as lower survival of adults and juvenile recruitment or decreased adult fitness, could be investigated in the future.

So far, the only study investigating intra‐seasonal variation in zooplankton availability, chick diet and breeding performance in little auks breeding in two large Svalbard colonies in Hornsund and Magdalenefjorden has revealed that late breeders in Magdalenefjorden were apparently mismatched regarding preferred food availability (Jakubas, Iliszko, et al., 2016). Despite the low‐energy content of food delivered to chicks late in the season, reproductive output and chick pre‐fledging mass were not affected significantly by the hatching date (Jakubas, Iliszko, et al., 2016). In contrast, our results demonstrate consistent within‐year effects of breeding timing, with chicks hatching earlier than the annual mean exhibiting faster growth and higher survival across colonies. This pattern indicates directional selection favouring earlier breeding within years, consistent with findings by Ribeiro et al. (2024), who showed that relative breeding timing was negatively associated with chick growth rate in little auks, indicating fitness consequences of phenology. Such consistent fitness advantages for earlier breeders suggest that ongoing environmental change may impose selective pressures favouring earlier breeding. However, as a long‐lived seabird, the little auk may exhibit limited capacity for rapid evolutionary adaptation, and phenological shifts are therefore more likely to reflect plastic responses rather than genetic change.

### 4.1 Future prospects

The ongoing and predicted warming of the Arctic climate is leading to higher air temperatures and widespread sea ice loss (IPCC, 2023). Sea ice loss alters light, evaporation, and precipitation, contributing to reduced snow and earlier melt (Bintanja, 2018; Bintanja & Selten, 2014). This, in turn, results in greater atmospheric moisture content and elevated precipitation levels. In maritime regions, where winter temperatures are increasingly crossing the freezing threshold, a larger proportion of this precipitation falls as rain rather than snow (Bintanja & Andry, 2017). This shift toward liquid precipitation contributes to reduced winter snow accumulation and an earlier onset of snowmelt in spring over the next decades. Our findings suggest that this earlier snowmelt is associated with an earlier onset of little auk breeding, though the extent and consistency of this response may vary across Arctic regions in the future.

Spatial heterogeneity remains a key feature of Arctic climate dynamics. The moderate changes observed in East Greenland, compared to Svalbard, suggest that this region could act as a future climate refuge for the little auks, which are tightly linked to ice conditions. Across all regions and climate scenarios, our results indicate that the most substantial decrease in snow cover occurs in the autumn months, reflecting a delay in the return of snow. This effectively extends the length of the snow‐free season—widening the Arctic's productive window primarily by shifting its end later into the year. Whether this extended autumnal period could benefit little auks remains doubtful, as they are not strongly time‐constrained during late summer. However, species which remain in the Arctic well into autumn (e.g. sandpipers: Ruthrauff et al., 2021) may gain more from a prolonged snow‐free season, a possibility that merits further ecological investigation.

In summary, our study demonstrates that snowmelt timing is a key and consistent driver of breeding phenology in little auks across multiple Arctic populations, with earlier snowmelt enabling earlier breeding. Earlier hatching within years was associated with improved chick growth and survival, indicating consistent directional selection favouring earlier breeding. However, interannual shifts toward earlier mean breeding were linked to improved survival in only one colony, highlighting spatial heterogeneity in the demographic consequences of phenological change. From a conservation perspective, these findings suggest that while some populations may initially benefit from earlier snowmelt, continued warming, regional differences in climate trajectories, and potential limits to phenological plasticity could pose risks to long‐term population viability. Understanding when and where these limits are reached will be critical for predicting the resilience of this key Arctic species in a rapidly changing climate.

## AUTHOR CONTRIBUTIONS

Martyna Syposz, Øystein Varpe, Tom S. L. Versluijs, Nomikos Skyllas, and Katarzyna Wojczulanis‐Jakubas conceived the ideas and designed the methodology. Martyna Syposz, Sébastien Descamps, Jérôme Fort, David Grémillet, Ann Harding, Dariusz Jakubas, Dorota Kidawa, Hallvard Strøm, and Katarzyna Wojczulanis‐Jakubas collected and curated the data. Martyna Syposz, Øystein Varpe, Sébastien Descamps, Nomikos Skyllas, Tom S.L. Versluijs, and Katarzyna Wojczulanis‐Jakubas analysed the data. Martyna Syposz led the writing of the manuscript. All authors contributed critically to the drafts and gave final approval for publication.

## CONFLICT OF INTEREST STATEMENT

The authors declare that they have no conflict of interest.

## Supporting information


**Table S1.** Median, mean and standard deviation of hatching date for each location and year with corresponding sample size (number of nests).
**Figure S1.** Density of hatching dates per year and location. The *x*‐axis shows day of year, and the *y*‐axis shows kernel density estimates. Colours indicate colonies.
**Figure S2.** Relationship between median hatching date and median laying date in Little Auks breeding in Hornsund. Line represents significant linear fit (Spearman's correlation coefficient *r* = 0.99).
**Table S2.** Central coordinates, area sizes (m^2^) and length × width (L × W, m) of colony polygons used for generating snowmelt dates.
**Figure S3.** Annual NDSI time series (DOY—day of year) per study site. Black points show daily NDSI; the solid black curve is the final GAM fit after outlier removal. The red point indicates the first day the smoothed curve dropped below NDSI threshold of 0.4 used to define snowmelt.
**Figure S4.** The results of snowmelt day against year for two satellite products: MODIS MOD09GA (black) and Sentinel‐2 L2A (red).
**Figure S5.** Correlation between snowmelt dates derived from MODIS (MOD09GA product) and Sentinel‐2 (L2A product) satellite imagery. Dashed lines indicate a perfect correlation (i.e. the estimated dates of snowmelt are identical for MODIS and Sentinel‐2). The red lines indicate the observed relationship between both satellite products, depicted as fits of linear models.

## Data Availability

Data available from the Dryad Digital Repository: https://doi.org/10.5061/dryad.j0zpc86wz (Syposz et al., 2026).
