# Supplementary material for: Snowmelt predicts earlier breeding across the latitudinal range of an Arctic nesting seabird, the Little Auk (Alle alle)
Source: J Anim Ecol. 2026 Jun 3;95(7):1248–59. doi: 10.1111/1365-2656.70287 (PMC13322177; doi:10.1111/1365-2656.70287)
Supplement: Supplementary file 1 — Table S1. Median, mean and standard deviation of hatching date for each location and year with corresponding sample size (number of nests). Figure S1. Density of hatching dates per year and location. The x‐axis shows day of year, and the y‐axis shows kernel density estimates. Colours indicate colonies. Figure S2. Relationship between median hatching date and median laying date in Little Auks breeding in Hornsund. Line represents significant linear fit (Spearman's correlation coefficient r = 0.99). Table S2. Central coordinates, area sizes (m2) and length × width (L × W, m) of colony polygons used for generating snowmelt dates. Figure S3. Annual NDSI time series (DOY—day of year) per study site. Black points show daily NDSI; the solid black curve is the final GAM fit after outlier removal. The red point indicates the first day the smoothed curve dropped below NDSI threshold of 0.4 used to define snowmelt. Figure S4. The results of snowmelt day against year for two satellite products: MODIS MOD09GA (black) and Sentinel‐2 L2A (red). Figure S5. Correlation between snowmelt dates derived from MODIS (MOD09GA product) and Sentinel‐2 (L2A product) satellite imagery. Dashed lines indicate a perfect correlation (i.e. the estimated dates of snowmelt are identical for MODIS and Sentinel‐2). The red lines indicate the observed relationship between both satellite products, depicted as fits of linear models. [file JANE-95-1248-s001.docx]

Supplementary Material

Snowmelt predicts earlier breeding across the latitudinal range of an Arctic nesting seabird, the Little Auk (*Alle alle*)

Martyna Syposz, Øystein Varpe, Sébastien Descamps, Jérôme Fort, David Grémillet, Ann Harding, Dariusz Jakubas, Dorota Kidawa, Nomikos Skyllas, Hallvard Strøm, Tom S.L. Versluijs, Katarzyna Wojczulanis-Jakubas

[*Supplementary material 1.* The median hatching date 2](#_Toc224397692)

[*Supplementary material 2.* Relationship between egg laying and hatching date 5](#_Toc224397693)

[*Supplementary material 3.* Polygons used for generating snowmelt day 6](#_Toc224397694)

[*Supplementary material 4.* Estimating snowmelt dates from NDSI using two-step outlier filtering and GAMs 7](#_Toc224397695)

[*Supplementary material 5.* Comparing snowmelt estimates based on MODIS and Sentinel-2 satellite products. 20](#_Toc224397696)

[References 23](#_Toc224397697)

## *Supplementary material 1.* The median hatching date

Table S1. Median, mean and standard deviation of hatching date for each location and year with corresponding sample size (number of nests).

| Location | Year | No. of nests | Median hatching date | Median hatching date (Day of year) | Mean hatching date (Day of year) | SD hatching date (Day of year) |
| --- | --- | --- | --- | --- | --- | --- |
| Bjørnøya | 2006 | 49 | 08/07/2006 | 189 | 188.9 | 1.6 |
| Bjørnøya | 2007 | 67 | 16/07/2007 | 197 | 197.2 | 2.5 |
| Bjørnøya | 2008 | 44 | 17/07/2008 | 199 | 198.6 | 2.8 |
| Bjørnøya | 2009 | 48 | 10/07/2009 | 191 | 190.6 | 2.6 |
| Bjørnøya | 2010 | 45 | 08/07/2010 | 189 | 189.1 | 2 |
| Bjørnøya | 2011 | 44 | 12/07/2011 | 193 | 193.6 | 2.6 |
| Bjørnøya | 2012 | 45 | 14/07/2012 | 196 | 196.5 | 3.1 |
| Bjørnøya | 2013 | 43 | 08/07/2013 | 189 | 189.2 | 2.1 |
| Bjørnøya | 2014 | 38 | 15/07/2014 | 196 | 197.1 | 2.3 |
| Bjørnøya | 2015 | 46 | 17/07/2015 | 198 | 198.6 | 3.4 |
| Bjørnøya | 2016 | 47 | 06/07/2016 | 188 | 187.7 | 2.9 |
| Bjørnøya | 2017 | 45 | 18/07/2017 | 199 | 199.3 | 2.2 |
| Bjørnøya | 2018 | 38 | 06/07/2018 | 187 | 187.3 | 2.5 |
| Bjørnøya | 2019 | 41 | 13/07/2019 | 194 | 194.5 | 1.9 |
| Bjørnøya | 2020 | 45 | 13/07/2020 | 195 | 194.5 | 2.1 |
| Bjørnøya | 2021 | 47 | 17/07/2021 | 198 | 197.4 | 2 |
| Bjørnøya | 2022 | 47 | 12/07/2022 | 193 | 193.1 | 2.7 |
| Bjørnøya | 2023 | 48 | 07/07/2023 | 188 | 188.5 | 3 |
| Bjørnøya | 2024 | 47 | 12/07/2024 | 194 | 194 | 2.2 |
| Hornsund | 2004 | 26 | 18/07/2004 | 200 | 199.4 | 3.2 |
| Hornsund | 2006 | 30 | 11/07/2006 | 192 | 192.6 | 1.7 |
| Hornsund | 2007 | 84 | 18/07/2007 | 199 | 201 | 4.2 |
| Hornsund | 2008 | 46 | 17/07/2008 | 199 | 200 | 3.4 |
| Hornsund | 2009 | 60 | 16/07/2009 | 197 | 197.4 | 2 |
| Hornsund | 2010 | 62 | 16/07/2010 | 197 | 198.3 | 1.8 |
| Hornsund | 2011 | 116 | 15/07/2011 | 196 | 196.4 | 2.5 |
| Hornsund | 2012 | 18 | 18/07/2012 | 200 | 199.6 | 1.1 |
| Hornsund | 2013 | 26 | 14/07/2013 | 195 | 195.3 | 1 |
| Hornsund | 2014 | 49 | 18/07/2014 | 199 | 198.3 | 1.1 |
| Hornsund | 2015 | 46 | 15/07/2015 | 196 | 197 | 1.9 |
| Hornsund | 2016 | 84 | 11/07/2016 | 193 | 193.4 | 2.2 |
| Hornsund | 2017 | 48 | 17/07/2017 | 198 | 198.2 | 2.1 |
| Hornsund | 2018 | 50 | 11/07/2018 | 192 | 192.3 | 2.1 |
| Hornsund | 2019 | 36 | 17/07/2019 | 198 | 197.8 | 1.8 |
| Hornsund | 2020 | 63 | 10/07/2020 | 192 | 193.2 | 3 |
| Hornsund | 2021 | 73 | 19/07/2021 | 200 | 199.7 | 1.7 |
| Hornsund | 2022 | 111 | 13/07/2022 | 194 | 193.5 | 2.9 |
| Hornsund | 2023 | 128 | 14/07/2023 | 195 | 195.7 | 1.9 |
| Hornsund | 2024 | 56 | 17/07/2024 | 199 | 199.4 | 2.1 |
| Isfjorden | 2005 | 17 | 12/07/2005 | 193 | 194.7 | 3.4 |
| Isfjorden | 2006 | 20 | 01/07/2006 | 182 | 183.2 | 3.3 |
| Isfjorden | 2009 | 25 | 13/07/2009 | 194 | 192.4 | 2.3 |
| Isfjorden | 2010 | 34 | 06/07/2010 | 187 | 188.6 | 4.5 |
| Isfjorden | 2011 | 24 | 11/07/2011 | 192 | 192.1 | 4.6 |
| Isfjorden | 2012 | 37 | 10/07/2012 | 192 | 192.7 | 2.4 |
| Isfjorden | 2013 | 21 | 11/07/2013 | 192 | 192 | 2.4 |
| Isfjorden | 2014 | 24 | 09/07/2014 | 190 | 190.7 | 2.2 |
| Isfjorden | 2015 | 16 | 08/07/2015 | 189 | 189 | 2.6 |
| Isfjorden | 2016 | 17 | 05/07/2016 | 187 | 187.6 | 1.9 |
| Isfjorden | 2017 | 15 | 12/07/2017 | 193 | 193.3 | 3 |
| Isfjorden | 2018 | 10 | 03/07/2018 | 184 | 185.2 | 1.9 |
| Ukaleqarteq | 2005 | 35 | 16/07/2005 | 197 | 197.1 | 2.8 |
| Ukaleqarteq | 2006 | 43 | 18/07/2006 | 199 | 198.8 | 2.7 |
| Ukaleqarteq | 2007 | 43 | 17/07/2007 | 198 | 198.8 | 3.3 |
| Ukaleqarteq | 2010 | 13 | 18/07/2010 | 199 | 198.8 | 2.3 |
| Ukaleqarteq | 2011 | 33 | 24/07/2011 | 205 | 204.8 | 3.2 |
| Ukaleqarteq | 2012 | 19 | 16/07/2012 | 198 | 198.5 | 1.7 |
| Ukaleqarteq | 2013 | 34 | 18/07/2013 | 199 | 199.4 | 2 |
| Ukaleqarteq | 2014 | 27 | 19/07/2014 | 200 | 199.4 | 2 |
| Ukaleqarteq | 2015 | 32 | 21/07/2015 | 202 | 202.8 | 2.2 |
| Ukaleqarteq | 2016 | 36 | 14/07/2016 | 196 | 196.7 | 2 |
| Ukaleqarteq | 2017 | 29 | 22/07/2017 | 203 | 204.1 | 2.1 |
| Ukaleqarteq | 2018 | 39 | 21/07/2018 | 202 | 201.7 | 3.5 |
| Ukaleqarteq | 2019 | 34 | 18/07/2019 | 199 | 199.3 | 2.1 |
| Ukaleqarteq | 2020 | 28 | 15/07/2020 | 197 | 197.5 | 2.5 |
| Ukaleqarteq | 2021 | 18 | 13/07/2021 | 194 | 194.8 | 3 |
| Ukaleqarteq | 2022 | 33 | 16/07/2022 | 197 | 197.8 | 2.4 |
| Ukaleqarteq | 2023 | 28 | 21/07/2023 | 202 | 202.5 | 3.1 |
| Ukaleqarteq | 2024 | 35 | 23/07/2024 | 205 | 205 | 1.8 |


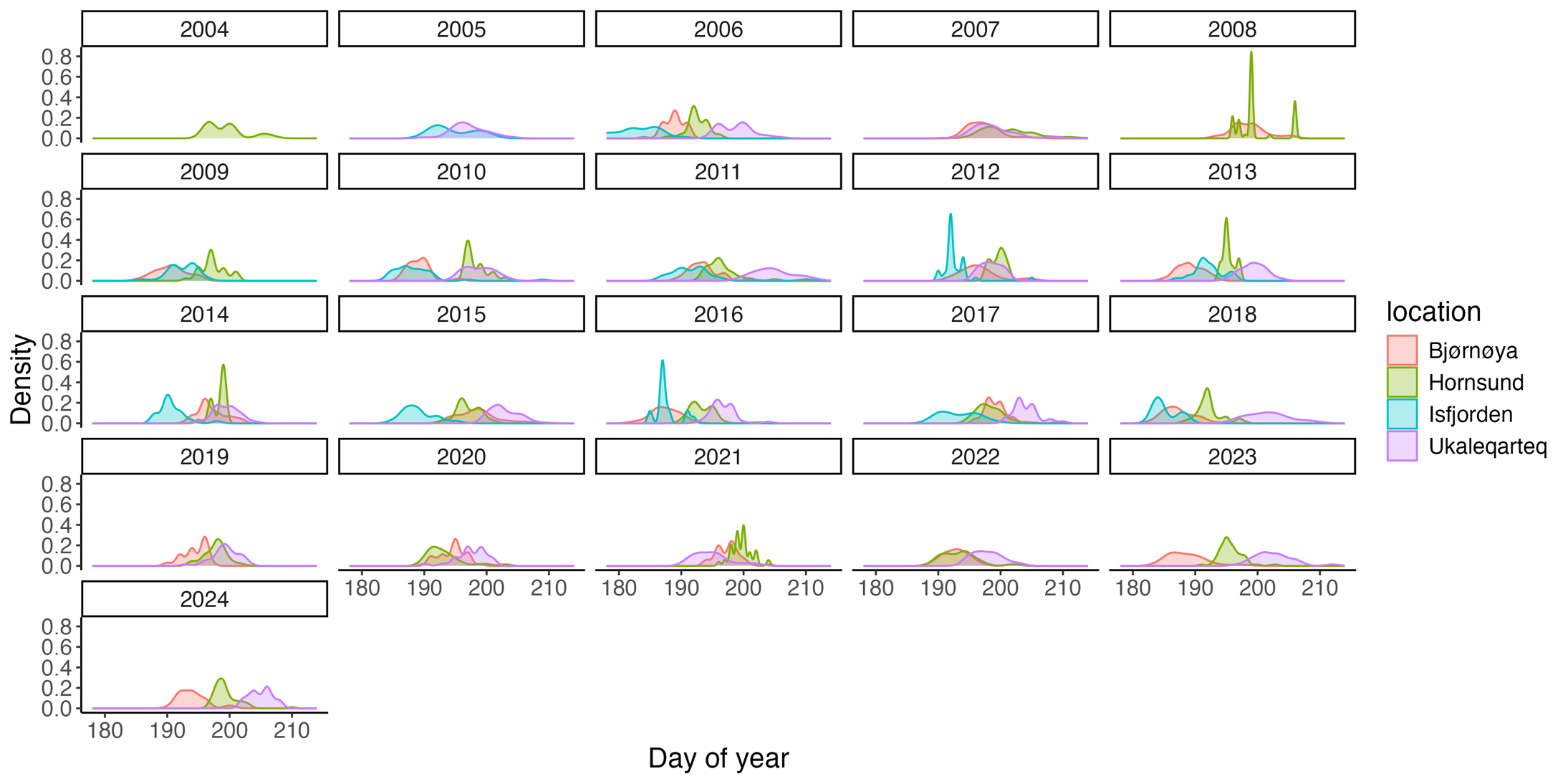


Figure S1. Density of hatching dates per year and location. The x-axis shows day of year, and the y-axis shows kernel density estimates. Colors indicate colonies.

## *Supplementary material 2.* Relationship between egg laying and hatching date

The data for both, laying and hatching date was available for 269 birds for 6 years in Hornsund colony (2006, 2011, 2012, 2019, 2021 & 2025). Correlating the seasonal medians of laying and hatching dates showed a strong, significant relationship (Spearman’s r = 0.99, p<0.001). To assess the robustness of this result given the small number of seasons (n = 6), we estimated post-hoc statistical power by simulating data under the observed effect size. Using 5,000 Monte Carlo replicates of a bivariate normal model with equivalent correlation and applying a Spearman correlation test, the estimated power at α = 0.05 (two-sided) was 0.89. We further calculated that Little Auks incubated an egg for an average ± SD 29 ± 1.3 days, i.e., similarly to 29 ± 0.8 days reported from Hornsund (Stempniewicz 1981).


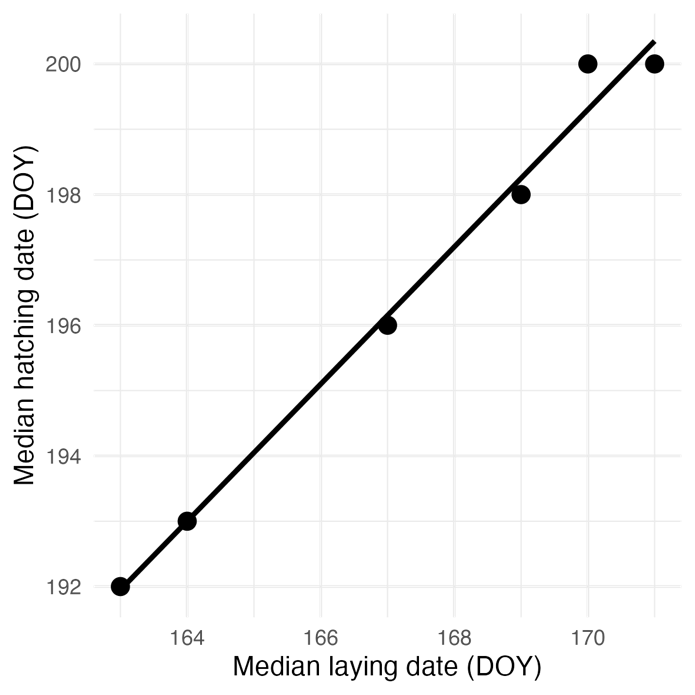


Figure S2. Relationship between median hatching date and median laying date in Little Auks breeding in Hornsund. Line represents significant linear fit (Spearman’s correlation coefficient r = 0.99).

## *Supplementary material 3.* Polygons used for generating snowmelt day

Table S2. Central coordinates, area sizes (m^2^) and length × width (L×W, m) of colony polygons used for generating snowmelt dates.

| Location | Central X | Central Y | Size (m^2^) | L×W |
| --- | --- | --- | --- | --- |
| Bjørnøya | 19.02264 | 74.37642 | 32 297.39 | 303 × 213 m |
| Hornsund | 15.52935 | 77.00937 | 21 472.26 | 209 × 186 m |
| Isfjorden | 15.34384 | 78.23102 | 172 813.7 | 599 × 346 m |
| Ukaleqarteq | -21.57416 | 70.72757 | 137 584.9 | 587 × 280 m |

## *Supplementary material 4.* Estimating snowmelt dates from NDSI using two-step outlier filtering and GAMs

To improve our estimates of the date of snowmelt, we applied a simple two-step procedure to detect and remove outliers while fitting Generalized Additive Models (GAMs) to the annual NDSI-timeseries. Such outliers could, for instance, result from false negatives in cloud detection. A two-step approach was essential because some data points might show large residuals solely due to the high leverage of extreme outliers in the initial GAM fit. By first removing these extreme outliers and then refitting the GAM, we could more reliably identify actual outliers. First, we fit a GAM to the raw NDSI-timeseries data and flagged all points with residuals exceeding 0.4 times the NDSI range for that pixel as outliers. We then refit the GAM, excluding these flagged points, and marked all remaining points with residuals exceeding 0.2 times the NDSI range as outliers. The remaining data were then used to fit a final GAM used for detecting the moment NDSI dropped below 0.4. For both steps in the outlier filtering process, we kept the GAM smoothing parameter ‘k’ at its default value of 10. For the final GAM (after outlier removal), we increased ‘k’ to 25 to allow a more flexible fit to the NDSI data, thereby improving the accuracy of the snowmelt date estimates. All GAMs were fitted using restricted maximum likelihood (REML).

Figure S3. Annual NDSI time series (DOY – day of year) per study site. Black points show daily NDSI; the solid black curve is the final GAM fit after outlier removal. The red point indicates the first day the smoothed curve dropped below NDSI threshold of 0.4 used to define snowmelt.

## *Supplementary material 5.* Comparing snowmelt estimates based on MODIS and Sentinel-2 satellite products.

Due to the relatively small size of our colonies, and the relatively coarse resolution of the MODIS satellite product (500x500m), our snowmelt estimates are based on one- to three pixels per study area. This might exacerbate biases in snowmelt estimates based on site-specific geographic features. For instance, the Ukaleqarteq colony is located on a north-facing cliff, resulting in persistent shading that may affect reflectance-based indices. Additionally, both the Ukaleqarteq and Isfjorden colonies are situated near the coastline, leading to mixed-pixel effects—a situation where a single pixel contains both land and ocean. These factors can significantly bias NDSI values and, consequently, the estimated dates of snowmelt.

To assess the potential impact of these limitations, and validate the robustness of our results, we conducted a parallel analysis using the higher-resolution Sentinel-2 level 2A product (L2A) (Main-Knorn *et al.* 2017).The analysis was again conducted in Google Earth Engine (Gorelick *et al.* 2017) using the R-package RGEE (Aybar *et al.* 2020) and automated workflows by Versluijs (2025). All chosen parameters and steps in the analysis were consistent with the analysis based on the MODIS product, except for the following differences: cloud filtering was conducted based on the S2 cloud probability dataset (s2cloudless, (Zupanc 2017), using a cloud probability threshold of 70%, and a spatial resolution of 150 meters to speed up computation time. Small cloud patches (false positives) were removed by eroding the binary cloud mask by 250 meters, and false negatives at the edges of the mask were reduced by dilating the resulting mask by 270 meters (Baetens, Desjardins & Hagolle 2019). Images with more than 75% cloud cover were excluded from the analysis and all remaining cloud-labelled pixels were masked. The date of snowmelt was again calculated using a threshold (NDSI<0.4) approach by fitting GAMs to annual NDSI-timeseries. NDSI was calculated as the normalized difference between the green (band 3) and short-wave infrared bands (band 11), but as the Sentinel-2 SWIR band has a lower spatial resolution (20 m) than the green band (10 m) we first used bicubic interpolation to resample the resolution of the green band to 20 meters. The higher spatial resolution of the Sentinel-2 product compared to MODIS (20 m vs 500 m) substantially reduced the likelihood of biases due to mixed-pixels and improves detection of fine-scale snow cover changes.

Results from Bjørnøya showed strong agreement between MODIS and Sentinel-2 estimates, with nearly identical snowmelt dates across years (**Fig. S4**). However, discrepancies were evident for other sites, most notably at the Ukaleqarteq colony, where MODIS and Sentinel-2 estimates differed by up to 35–40 days, indicating a substantial impact of site-specific geographic features on the snowmelt estimates.

Despite these absolute differences, the interannual variability in snowmelt patterns between the two datasets is consistent. To quantify this, we conducted correlation analyses between annual snowmelt dates estimated by both satellites for the period 2019–2024. Correlation plots confirm that while absolute estimates differ for some sites, the relative interannual trends are preserved (Pearson's correlation coefficients: Bjørnøya: r = 0.98, Hornsund: r = 0.76, Isfjorden: r = 0.99, Ukaleqarteq: r = 0.79, **Fig. S5**).

These results suggest that although MODIS may not provide accurate absolute snowmelt dates in some locations due to constraints in the spatial resolution of the satellite product, it still reliably captures the relative variation in snowmelt timing across years for our study sites. This is particularly evident in the consistency of the slope in correlation plots, which indicates that temporal trends (i.e., early vs. late years) are robust to the choice of satellite product. Since our analysis of phenological responses focuses on interannual variability rather than absolute dates, the influence of these MODIS-based discrepancies is minimal.


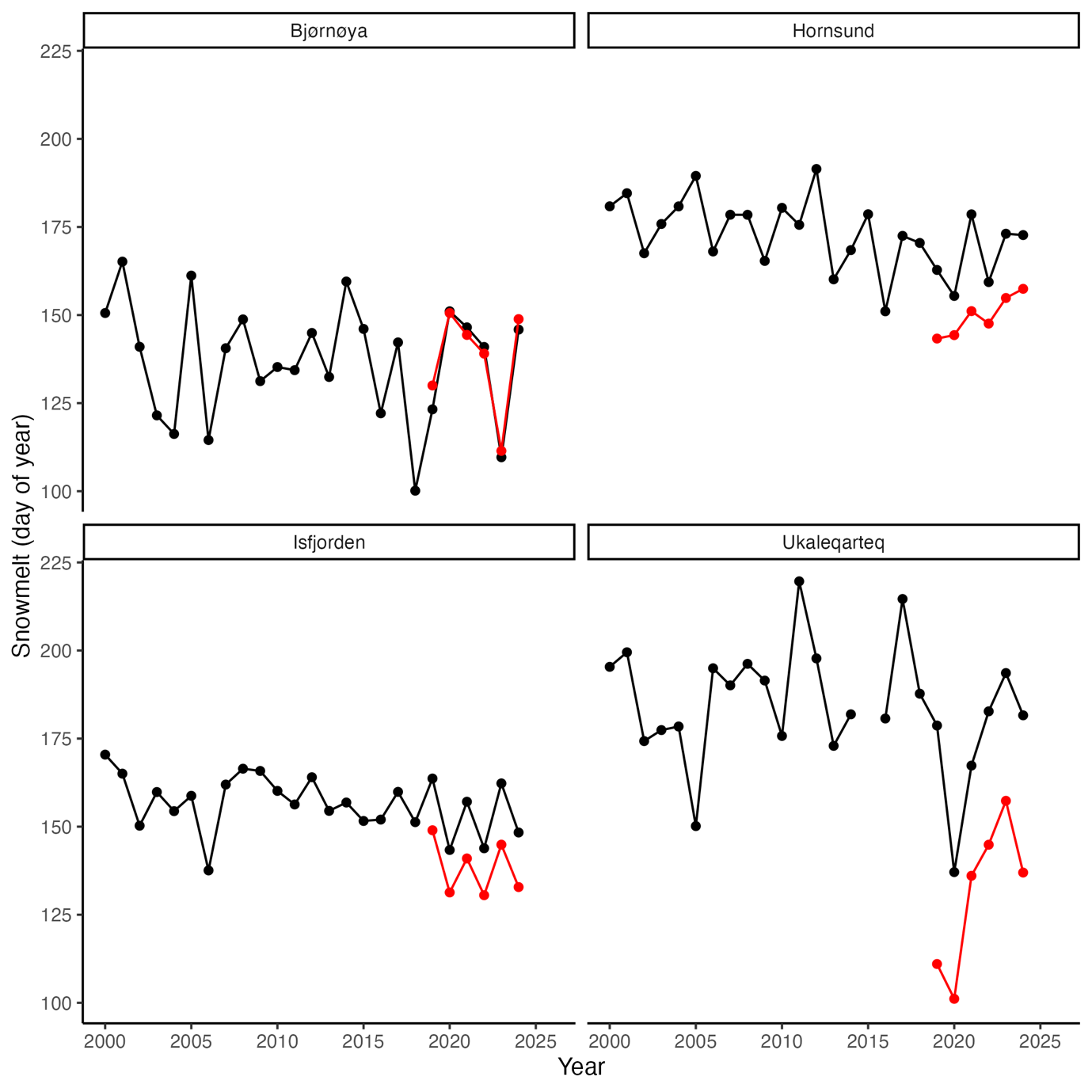


Figure S4. The results of snowmelt day against year for two satellite products: MODIS MOD09GA (black) and Sentinel-2 L2A (red).


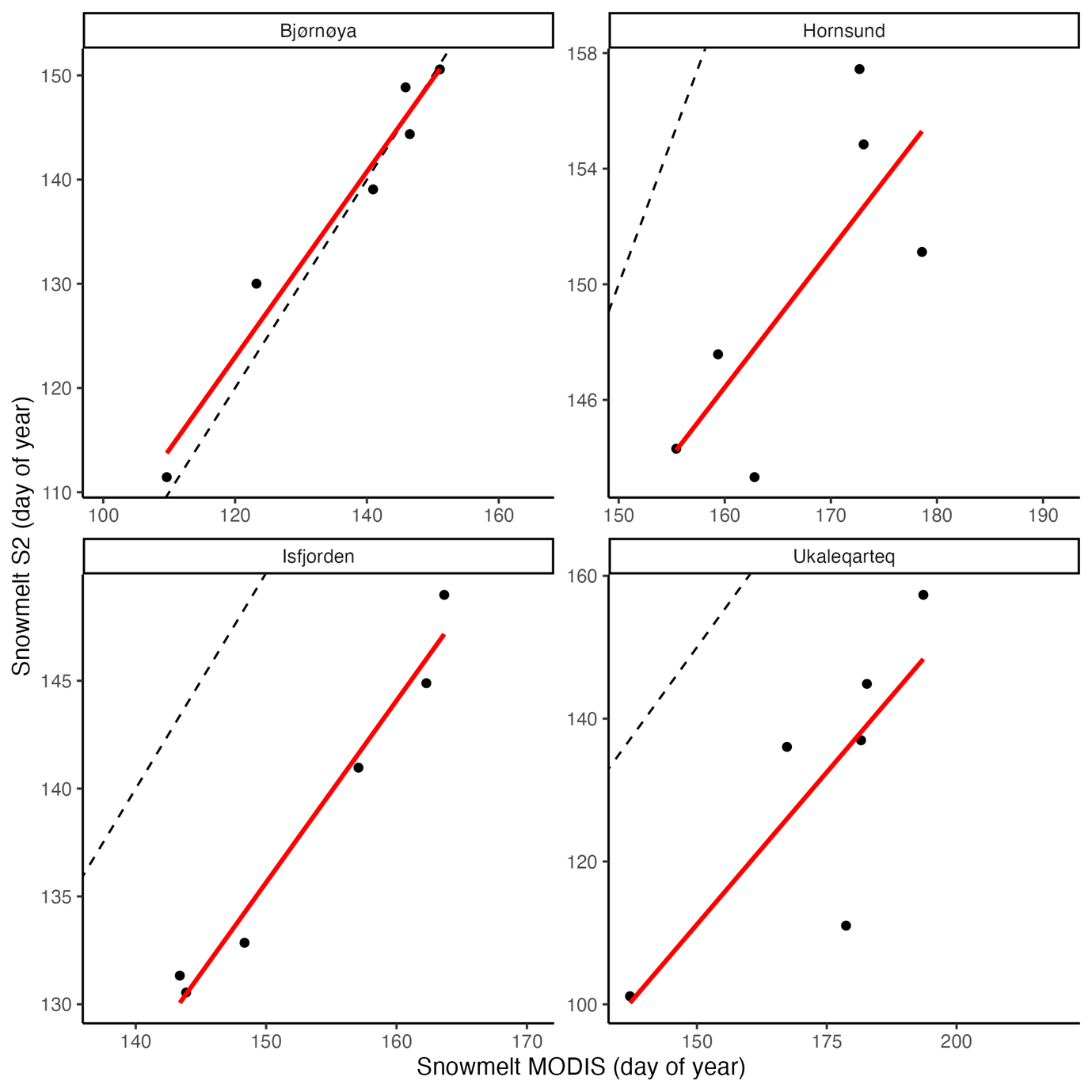


Figure S5. Correlation between snowmelt dates derived from MODIS (MOD09GA product) and Sentinel-2 (L2A product) satellite imagery. Dashed lines indicate a perfect correlation (i.e. the estimated dates of snowmelt are identical for MODIS and Sentinel-2). The red lines indicate the observed relationship between both satellite products, depicted as fits of linear models.

##

## References

Aybar, C., Wu, Q., Bautista, L., Yali, R. & Barja, A. (2020) rgee: An R package for interacting with Google Earth Engine. *Journal of Open Source Software,* **5,** 2272.

Baetens, L., Desjardins, C. & Hagolle, O. (2019) Validation of Copernicus Sentinel-2 Cloud Masks Obtained from MAJA, Sen2Cor, and FMask Processors Using Reference Cloud Masks Generated with a Supervised Active Learning Procedure. *Remote Sensing,* **11,** 433.

Gorelick, N., Hancher, M., Dixon, M., Ilyushchenko, S., Thau, D. & Moore, R. (2017) Google Earth Engine: Planetary-scale geospatial analysis for everyone. *Remote Sensing of Environment,* **202,** 18-27.

Main-Knorn, M., Pflug, B., Louis, J., Debaecker, V., Müller-Wilm, U. & Gascon, F. (2017) *Sen2Cor for Sentinel-2*. SPIE.

Stempniewicz, L. (1981) Breeding biology of the little auk *Plautus alle* in the Hornsund region, Spitsbergen. *Acta ornithologica,* **18**.

Versluijs, T.S.L. (2025) RGEE_Snowmelt (v1.3.0). Zenodo: <https://doi.org/10.5281/zenodo.8229031>.

Zupanc, A. (2017) Improving cloud detection with machine learning.
